# Highly Loaded Behavior of Kinesins Increases the Robustness of Transport Under High Resisting Loads

**DOI:** 10.1371/journal.pcbi.1003981

**Published:** 2015-03-03

**Authors:** Woochul Nam, Bogdan I. Epureanu

**Affiliations:** Department of Mechanical Engineering, University of Michigan, Ann Arbor, Michigan, United States of America; Harvard University, UNITED STATES

## Abstract

Kinesins are nano-sized biological motors which walk by repeating a mechanochemical cycle. A single kinesin molecule is able to transport its cargo about 1 μm in the absence of external loads. However, kinesins perform much longer range transport in cells by working collectively. This long range of transport by a team of kinesins is surprising because the motion of the cargo in cells can be hindered by other particles. To reveal how the kinesins are able to accomplish their tasks of transport in harsh intracellular circumstances, stochastic studies on the kinesin motion are performed by considering the binding and unbinding of kinesins to microtubules and their dependence on the force acting on kinesin molecules. The unbinding probabilities corresponding to each mechanochemical state of kinesin are modeled. The statistical characterization of the instants and locations of binding are captured by computing the probability of unbound kinesin being at given locations. It is predicted that a group of kinesins has a more efficient transport than a single kinesin from the perspective of velocity and run length. Particularly, when large loads are applied, the leading kinesin remains bound to the microtubule for long time which increases the chances of the other kinesins to bind to the microtubule. To predict effects of this behavior of the leading kinesin under large loads on the collective transport, the motion of the cargo is studied when the cargo confronts obstacles. The result suggests that the behavior of kinesins under large loads prevents the early termination of the transport which can be caused by the interference with the static or moving obstacles.

## Author Summary

Kinesins are molecular motors which work when they bind to tracks called microtubules (MTs). In a cell, cargoes of kinesins are transported to very distant destinations compared to a short transport ability of single kinesins. To study this difference, we establish a novel quantitative model capable of capturing the transport by teams of kinesins. While an entire team does not completely dissociate from a MT easily, individual kinesins continuously bind and unbind from MTs. Because the binding occurs faster than the unbinding process, the transported distance increases significantly over the number of kinesins in the team. Another barrier on cellular transport is the disturbance to the motion of the cargo by other cytoplasmic particles. Thus, we also consider the effect of cellular particles by exerting temporary high resisting loads on the cargo. Our model predicts that kinesins remain bound on the MT for a long time when the high resisting loads act on them. This behavior increases the probability that other unbound kinesins rebind to MTs. Consequently, more kinesins can participate in the transport. Then, the cargo is able to overcome interruption by other cellular particles. Thus, a robust intracellular transport can be realized by this mechanism.

## Introduction

Kinesins move 8 nm per step [1–4] along MT by using energy obtained from ATP hydrolysis [5–7]. Among the kinesin superfamily, this study focuses on kinesin–1 (referred to simply as kinesin) which has two identical heads [8, 9]. Several experiments have measured the run length of cargoes transported by kinesins. Block et al. [10] reported that cargoes move about 1.4 *μ*m when they are pulled on average by about one and half kinesin molecules. The distribution of the run length in those experiments follows an exponential probability distribution. The effects of external resisting loads exerted on the cargo and the concentration of ATP on the run length was observed by Schnitzer et al. [11]. The run length decreases with increasing resisting load and decreasing ATP concentration. In the experiment performed by Uemura et al. [12, 13], the magnitude of the loads causing unbinding were measured. They exerted loads toward the plus or the minus end of the MTs to discover the effects of the direction of the load. Their results show that kinesins tend to unbind more easily when subjected to loads toward the plus end of the MTs than by loads toward the opposite direction. However, the difference is not considerable. The experiment of Beeg et al. [14] focused on the transport of cargoes by groups of kinesins. To observe the relation between the number of kinesins and the run length, they varied the number of kinesins attached to the cargo. The run length increased as more kinesins participate in the transport. However, the run length was surprisingly reduced when the cargo was moved by considerably many kinesins.

Mathematical models have been proposed to calculate the run length of kinesin. Schnitzer et al. [11] established an equation regarding the run length of a single kinesin molecule by using Arrhenius-Eyring kinetics. The approach produced a successful fit to experimental data for various ATP concentrations and external loads. However, the model is only applicable to the motion of single molecules. For transport by several kinesins, Klumpp et al. [15] utilized discrete Markov chains to obtain a master equation regarding the number of motors which effectively participate in the transport. Then, they obtained the stationary solution for the master equation. By substituting the transition rates of kinesins (i.e., binding rate to MT, and unbinding rate from MT), their model obtained an analytical solution for the mean value and the probability density function (pdf) of the run length. Their unbinding model accounts for the effects of load by assuming that the load is equally distributed over every kinesin bound on the MT. However, the distribution of loads over motors continuously changes due to the stochastic motion of kinesins [16–18]. Furthermore, their binding model is not able to capture the locations where rebinding occurs, despite the fact that those locations also affect the collective transport.

The goal of this study is to develop a binding/unbinding model which is able to capture the stochastic unbinding and binding of kinesins to the MTs and their dependencies on the force acting on them. By using the model, the run length and velocity of collective transport under constant loads are obtained. The characterization on the unbinding of kinesins captures an interesting behavior of kinesins, namely that they spend a long time remaining on the MT when large resisting loads are applied. The model predicts that this behavior of kinesin is beneficial for the cargo to overcome obstacles. Also, the velocity of collective transport is affected by the stochastic rebinding process as well as by the velocity of kinesin molecules itself.

## Models

**Fig 1:**
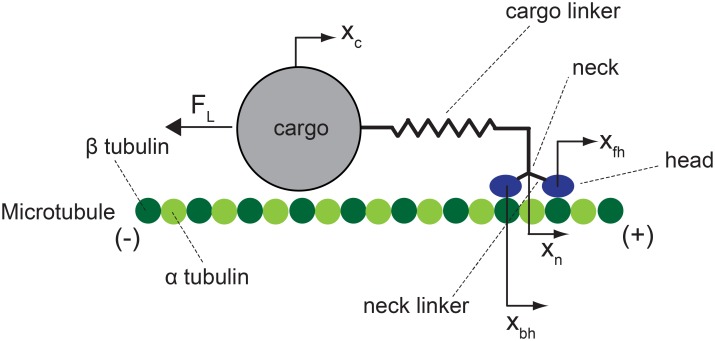
The mechanistic model of kinesin is schematically shown. The kinesin molecule is composed of two heads, two neck linkers, and a neck. The neck is connected to the outer surface of the cargo via a cargo linker. The small spheres conceptually represent *α* and *β* tubulin which form the MT. Kinesins walk from the minus end to the plus end of the MT. Coordinates *x*_*c*_, *x*_*n*_, *x*_*fh*_ and *x*_*bh*_ are the position of the cargo, that of the neck, and the positions of the forward and backward heads. *F*_*L*_ denotes the load acting on the cargo; its direction is along the MT. The sign of *F*_*L*_ is plus when it is toward the minus end of the MT.

**Fig 2:**
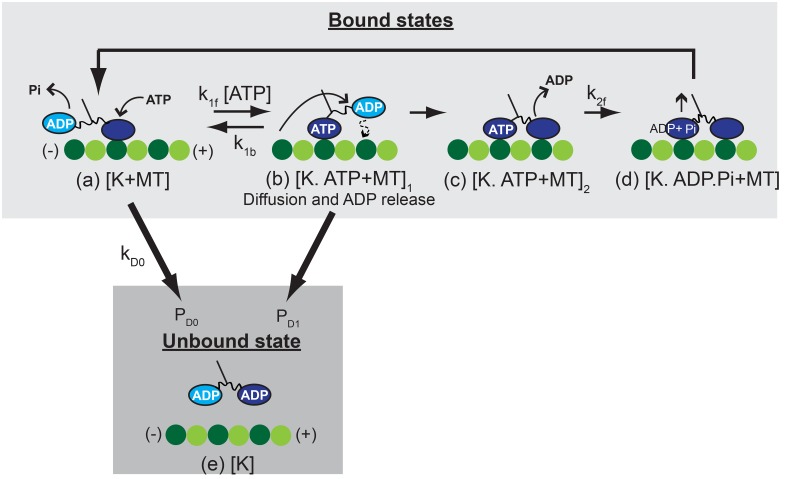
The kinesin cycle is depicted. The states in the upper box relate to the walking cycle of the kinesin. K denotes the kinesin molecule. The lower box relates to the unbound state of the kinesin. The variables denoted by k are transition rates between states, and *P*_*D*0_ and *P*_*D*1_ represent the probability of unbinding from the MT when the kinesin is in the state [K + MT] and [K.ATP + MT]_1_. (a) An ATP molecule binds to the leading head of the kinesin. (b) The binding of ATP to the kinesin head results in a structural changes in the head [38]. This change induces the docking of the neck linker to its head. The docking of the neck linker to the leading head generates a force to move the trailing head toward the plus end of MT. Then, the trailing head diffuses to the next binding site of MT by Brownian motion. (c) The moving head binds to the MT and releases ADP. (d) ATP in the rear head is hydrolyzed, and then this hydrolysis enables the release of phosphate (Pi) from the head. Then, the neck linker returns to the disordered state from the docked state.

The mechanistic model of a previous study [16, 19] is used to capture the walking motion of kinesins. The components of the mechanistic model are shown in Fig. 1. A kinesin walks by repeating a mechanochemical cycle; one cycle at every step, as shown in Fig. 2. Kinesin molecules are assumed to have state [K+MT], [K.ATP + MT]_1_, [K.ATP + MT]_2_, or [K.ADP.Pi + MT] when they are bound to the MT. Thus, before calculating the unbinding probability, the instant of the transition between bound states is captured by the mechanistic model. The details of the mechanistic model are provided in S1 Text. Both heads of kinesin are strongly bound during the state [K.ATP + MT]_2_ and [K.ADP.Pi + MT]. Thus, it is assumed that the probabilities to unbind during the states [K.ATP + MT]_2_ and [K.ADP.Pi + MT] are negligible, and the preponderance of instances of unbinding occurs either from state [K + MT] or state [K.ATP + MT]_1_. Thus, the unbinding probabilities corresponding to states [K + MT] and [K.ATP + MT]_1_ are calculated in this study.

Among several kinesins connected to a cargo, some kinesins walk along the MT, and others are not attached to the MT but just follow the cargo. Since both heads of unbound kinesins are free from the MT, the position of these heads fluctuates over time. The effect of this fluctuation on the rebinding is included in the model by calculating the pdf of the positions of unbound kinesins.

### Unbinding model

The unbinding probability during the state [K+MT] is calculated over time using the transition rate *k*_*D*0_ from the state [K+MT] to the unbound state [K], as shown in Fig. 2. If the *i* ^th^ cycle starts at an instant *t*_*i*_, the unbinding probability in state [K+MT] during this cycle is obtained by solving the following set of equations with the initial condition *P*_[*K*+*MT*]_(*t* = *t*_*i*_) = 1.

$$\begin{array}{c} \frac{d}{dt}P_{\left[K+MT\right]}=-k_{D0}P_{\left[K+MT\right]},\\ P_{\left[K+MT\right]}\left(t\right)+P_{D0}\left(t\right)=1, \end{array}$$ (1)

where *P*_[K+MT]_ is the probability that kinesin remains attached to the MT, and *P*_*D*0_(*t*) is the unbinding probability in state [K+MT]. Thus, the growth of *P*_*D*0_ over time can be calculated as

$$\begin{array}{c} P_{D0}\left(t\right)=1-\exp\left(-k_{D0}t\right) \end{array}$$ (2)

The time constant of the cargo motion transported by single kinesin was experimentally measured by Carter et al. [20] to be approximately 15.3 *μ*s for a resisting load of 5 pN applied to the cargo. The dwell time of kinesin has also been measured experimentally by numerous researchers for that load, including Visscher et al. [21]. That dwell time is about 70 ms. Hence, the duration of state [K.ATP + MT]_1_ is much shorter than the dwell time and thus negligibly short. Due to this very short duration of state [K.ATP + MT]_1_, a single unbinding probability value (i.e., *P*_*D*1_) is used for this state instead of capturing the changes of the probability over time.

This model is able to predict the instant of transition between bound states. Thus, the effect of ATP concentration on unbinding is intrinsic to the model. The model also accounts for the effects of force by using Bell model [22] and expressions inspired by Boltzmann’s law as

$$\begin{array}{cc} k_{D0}=k_{D0,0}\;\exp\;\frac{|F_{k}|d_{0}}{k_{B}T}, & \\ P_{D1}=P_{D1,0}\;\exp\;\frac{|F_{k}|d_{1}}{k_{B}T}, \end{array}$$ (3)

where *k*_*D*0, 0_, *P*_*D*1, 0_, *d*_0_, and *d*_1_ are parameters of the unbinding model. *F*_k_ is the force transferred from the cargo to the kinesin. The equation for this force is provided in S1 Text. The occurrence of an unbinding event is determined by comparing the calculated probability with uniformly distributed random numbers.

**Fig 3:**
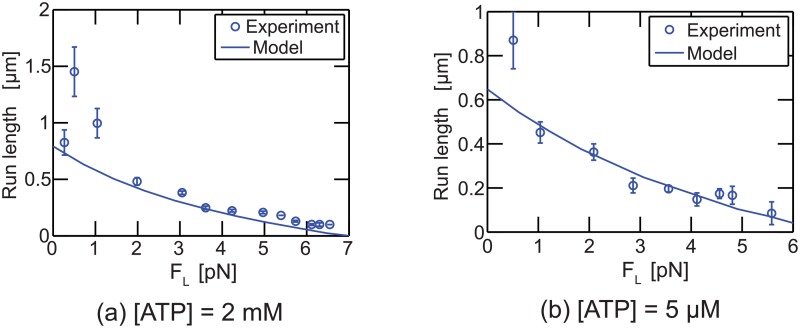
The run length of single kinesins is shown. (a) shows the run length for various loads for [ATP] = 2 mM; (b) shows the run length when [ATP] = 5 *μ*M.

The transport performed by a single kinesin does not include the rebinding process. Instead, the experimentally observed run lengths of single kinesins [11] are used to determine parameters of the unbinding model. The parameters of the model are obtained so that the model predicts run lengths measured experimentally. The fitting is done by using the nonlinear least-squares fit function (lsqnonlin) in MATLAB. Note that the results of the fitting indicate that the effects of the load on unbinding during the state [K + MT] are very weak. Hence, *d*_0_ is very small compared to other parameters. Thus, the value of *d*_0_ is set as zero. Table 1 represents the values of the parameters, and Fig. 3 shows the run length of the experiments and the model. The values of the parameters can be changed by the interactions between kinesins. When the number of kinesins is small (e.g., between 1 and 5) and not considerably large (like hundreds), the effect of interference among kinesins on the unbinding of kinesins is assumed negligible. Thus, the parameters of unbinding used in this study apply to the cargoes transported by one to five kinesins.

**Table 1:** The values of parameters regarding unbinding.

depth 6pt width 0pt Parameter	Value	Unit
*k*_*D*0, 0_	0.01633	s^−1^
*P*_*D*1, 0_	0.01	Probability
*d*_0_	0	nm
*d*_1_	1.272	nm

### Rebinding model

**Fig 4:**
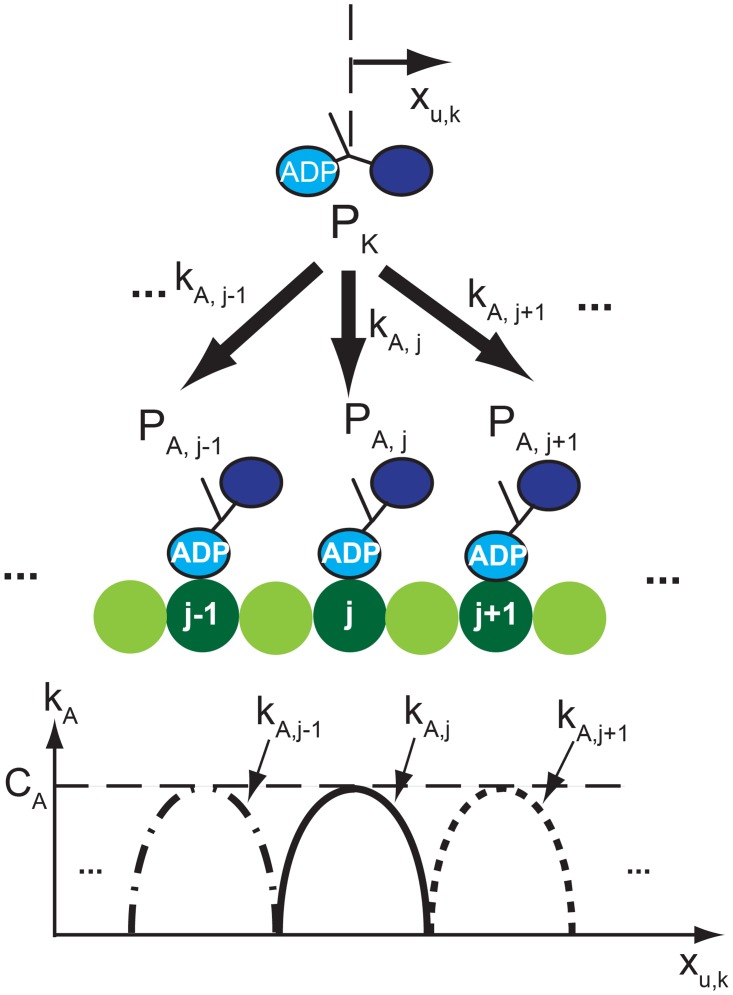
The rebinding of the unbound kinesin is depicted. The unbound kinesin has probabilities to rebind at several binding sites of the MT. *P*_*K*_ is the probability to stay in the unbound state, while *P*_*A*, *j*−1_, *P*_*A*, *j*_, and *P*_*A*, *j*+1_ are the probabilities to rebind at the (*j* − 1)^th^, *j* ^th^, and (*j* + 1)^th^ binding site. *k*_*A*, *j*_ is the transition rate from the unbound state to the bound state at the *j* ^th^ binding site. The values of *k*_*A*_*j*_−1_, *k*_*A*_*j*__, and *k*_*A*_*j*_+1_ vary over the position of unbound kinesin *x*_*u*, *k*_ with respect to the position of binding sites of the MT. *C*_*A*_ is a parameter of the model which denotes the rate of rebinding to a certain binding site when the unbound kinesin is exactly above that site.

The kinesin released from the MT can possibly bind again to several binding sites, as shown in Fig. 4. The probability of rebinding to the *j* ^th^ binding site is calculated using the transition rate *k*_*A*, *j*_. The value of *k*_*A*, *j*_ decreases as the distance between the unbound kinesin and the *j* ^th^ binding site increases. The position of the neck and heads of the unbound kinesins are assumed to be the same. Thus, those positions are denoted by a single variable *x*_*u*, *k*_. The dependence on the distance is assumed to have a parabolic distribution, as shown in Fig. 4.

Since the time scale of the thermal fluctuations of an unbound kinesin is very short, the value of *k*_*A*, *j*_ (which depends on the position of the unbound kinesin) also changes rapidly over time. Intensive computations are required to capture the value of *k*_*A*, *j*_ over time. Instead of calculating the rapidly changing *k*_*A*, *j*_, the following method is used. First, the pdf of the position of the unbound kinesin is obtained by using the strain energy in the kinesin structure. Then, the time average of *k*_*A*, *j*_ is obtained at every time step by spatially integrating the value of *k*_*A*, *j*_ weighted by the pdf of the unbound kinesins. By using this method, the amount of computation reduces significantly because the time step can be determined by the dynamics of bound kinesins not by the fluctuating motion of the unbound kinesin.

#### 

##### pdf of the position of the unbound kinesin

**Fig 5:**
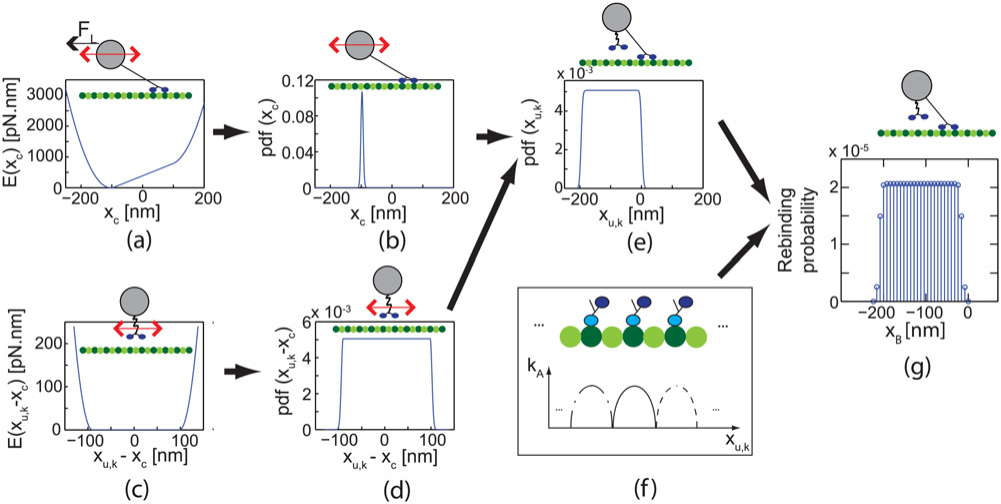
The rebinding model is schematically shown. The pdf of the position of the cargo pdf(*x*_*c*_) (b) is obtained from the strain energy in the structure of the bound kinesins (a). The pdf of the position of the unbound kinesin respect to the cargo pdf(*x*_*u*, *k*_ − *x*_*c*_) (d) is obtained from the strain energy in its structure (c). The pdf of the position of the unbound kinesin pdf(*x*_*u*, *k*_) (e) is calculated as the convolution of pdf(*x*_*c*_) and pdf(*x*_*u*, *k*_). (f) shows the values of *k*_*A*_ over the position of unbound kinesin. (g) The rebinding probability on each binding site during a time step is obtained by using *k*_*A*, *j*_ and pdf(*x*_*u*, *k*_).

The pdf of the position of the unbound kinesin is obtained by using two pdf of position (i.e., the pdf of the cargo and pdf of unbound kinesin with respect to the cargo). The pdf of the position of the cargo can be obtained using the Boltzmann law, *P* ∝ exp(−*E*/*k*_*B*_ *T*), where *E* is the mechanical potential energy in the kinesin. When only one kinesin is bound to the MT, the potential energy arisen from the tension in the bound kinesin is calculated as

$$\begin{array}{c} E_{c}\left(x_{c}\right)=\left\{\begin{array}{cc} \frac{1}{2}K_{e}{\left(x_{c}-x_{r}-L_{c}\right)}^{2}+F_{L}\left(x_{c}-<x_{c}>\right) & \text{if}\;x_{c}>x_{r}+L_{c},\\ \frac{1}{2}K_{e}{\left(x_{c}-x_{r}+L_{c}\right)}^{2}+F_{L}\left(x_{c}-<x_{c}>\right) & \text{if}\;x_{c}<x_{r}-L_{c},\\ F_{L}\left(x_{c}-<x_{c}>\right) & \text{otherwise,} \end{array}\right. \end{array}$$ (4)

where *E*_*c*_ is the potential energy of the bound kinesin, and $x_{r}=\frac{1}{2}(x_{fh}+x_{bh})$ is the position in the middle of two heads. $K_{e}=\frac{2K_{n}K_{c}}{2K_{n}+K_{c}}$ is the equivalent stiffness of kinesin due to its cargo linker and two neck linkers. *K*_*c*_ and *K*_*n*_ are the stiffness of the cargo linker and the neck linker. < *x*_*c*_ > is the position of the cargo where *F*_*L*_ and *F*_k_ are balanced. The potential energy of the cargo is shown in Fig. 5 (a). Using the spatial distribution of energy and the Boltzmann law, the pdf of the position of the cargo, pdf(*x*_*c*_ = *x*), is obtained, as shown in Fig. 5 (b). The pdf of the position of the unbound kinesin (*x*_*u*, *k*_) is obtained through two steps. First, the pdf of the unbound kinesin with respect to the cargo, pdf(*x*_*u*, *k*_ − *x*_*c*_ = *x*), is calculated by considering the potential energy of the cargo linker of the unbound kinesin, as shown in Figs. 5 (c) and (d). Note that this pdf is identical with the pdf of the cargo in the absence of the load. This pdf is also assumed as invariant with respect to external loads on the cargo because the external loads do not act on the unbound kinesins. Next, the pdf of the unbound kinesin, pdf(*x*_*u*, *k*_ = *x*), is determined by the convolution of two pdfs of *x*_*c*_ and *x*_*u*, *k*_ − *x*_*c*_ as

$$\begin{array}{c} \mathrm{pdf}\left(x_{u,k}=x\right)=\int_{-\infty }^{\infty }\mathrm{pdf}\left(x_{c}=u\right)\;\mathrm{pdf}\left(x_{u,k}-x_{c}=x-u\right)\;du. \end{array}$$ (5)

The pdf of the unbound kinesin is shown in Fig. 5 (e).

##### Time average of the transition rate *k*_*A*_

**Fig 6:**
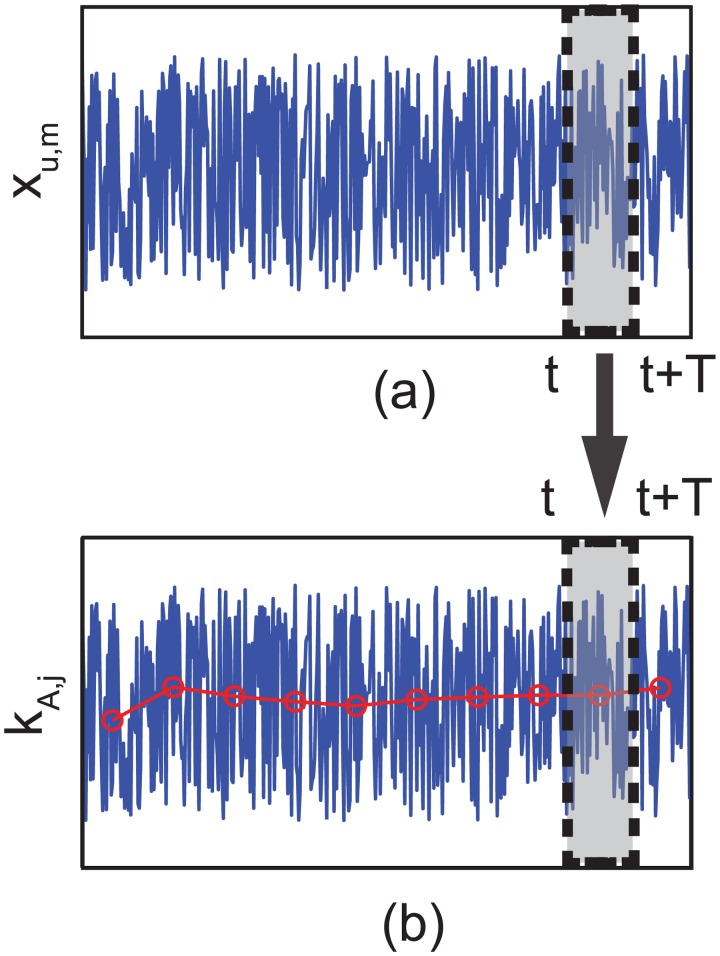
Time average of the transition rate is depicted. (a) shows the fluctuating position *x*_*u*, *k*_ of unbound kinesin, and (b) shows the transition rate *k*_*A*, *j*_ to rebind at the *j* ^th^ binding site. The rate also fluctuates over time. Circles and the bold line indicate the moving average of the rate.

Instead of calculating the rapidly changing value of *k*_*A*, *j*_ over time, the time average of the transition rate is used to avoid intensive computations with excessively short time steps for capturing the changes. The time average is calculated several times before a rebinding event so as to consider the changes in the position of the cargo by bound kinesins. This idea is depicted in Fig. 6. By using this approach, the probability *P*_*A*, *j*_(*t*, *t* + *T*) to rebind to the *j* ^th^ binding site between two time steps *t* and *t*+*T* can be expressed as

$$\begin{array}{c} P_{A,j}\left(t,t+T\right)=<k_{A,j}>P_{K}\left(t\right)\;T, \end{array}$$ (6)

where < *k*_*A*, *j*_ > is the time average of *k*_*A*, *j*_ between *t* and *t*+*T. P*_*K*_(*t*) is the probability that the kinesin remains in the unbound state until time is *t*.

**Fig 7:**
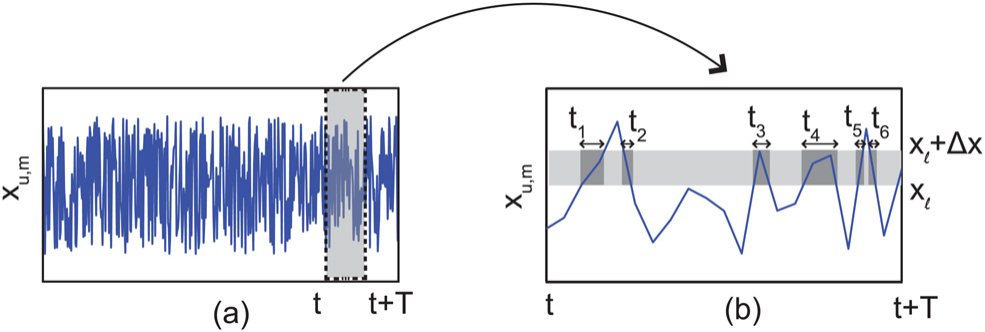
(a) shows the fluctuating position of unbound kinesin. (b) shows details for *x*_*u*, *k*_ between time steps *t* and *t* + *T. t*_1…6_ are the durations when *x*_*u*, *k*_ exists between *x*_ℓ_ and *x*_ℓ_ + Δ*x*.

The efficiency of the computation is improved by converting the time average < *k*_*A*, *j*_ > into a spatial average. First, by using the pdf of the unbound kinesin (i.e., pdf(*x*_*u*, *k*_ = *x*)), the time when *x*_*u*, *k*_ is between two points (*x*_ℓ_ and *x*_ℓ_ + Δ*x* in Fig. 7) during *t* and *t* + *T* can be calculated as

$$\begin{array}{c} \Delta \tau_{\ell }=T\int_{x_{\ell }}^{x_{\ell }+\Delta x}\mathrm{pdf}\left(x_{u,k}=x\right)\;dx. \end{array}$$ (7)

Then, the time average of *k*_*A*, *j*_ is changed to the spatial average as followings.

$$\begin{array}{c} <k_{A,j}>=\frac{1}{T}\int_{t}^{t+T}k_{A,j}\left(\tau \right)d\tau =\frac{1}{T}\lim_{\Delta t\to 0}\sum_{n}k_{A,j}\left(t_{n}\right)\Delta t\\ =\frac{1}{T}\lim_{\Delta x\to 0}\sum_{\ell }k_{A,j}\left(x_{\ell }\right)\Delta \tau_{\ell }=\int_{-\infty }^{\infty }k_{A,j}\left(x\right)\;\mathrm{pdf}\left(x_{u,k}=x\right)dx \end{array}$$ (8)

where pdf(*x*_*u*, *k*_ = *x*) is the pdf of the position of the unbound kinesin.

By using the pdf of the unbound kinesin and Equation 6, the probability that the kinesin rebinds to each binding site between two time steps can be obtained, as shown in Fig. 5 (g).

In vitro experiments shows that kinesins rebind to the MT with the rate of approximately 5 s^−1^ in average in the absence of load [14, 23, 24]. Using this information, the maximal value of *k*_*A*_ (which represents as *C*_*A*_ in Fig. 4) can be determined as 7.68 s^−1^. This value provides the average rebinding rate of 5.1 s^−1^ which is the rate reported in [14]. For various ATP concentrations and loads, the average rebinding rate remains almost the same. However, the rebinding probabilities over binding sites vary, as shown in Fig. 5 (g). The method to determine the instant and location of rebinding is provided in the following.

### Simulation procedure

The simulation begins with the state where one kinesin is bound to the MT. The dynamics of the cargo, the chemical reactions and the unbinding are considered for bound kinesins, and rebinding to the MT are considered for unbound kinesins at every time step. The positions of the necks of bound kinesins and cargo are obtained by using the mechanistic model (which is described in S1 Text). Then, the chemical states the bound kinesins are determined from the mechanistic model. At the beginning of every cycle of bound kinesin, the unbinding probability is zero. Four uniformly distributed random numbers (e.g., *r*_*w*_, *r*_*d*0_, *r*_*d*1_, and *r*_*b*_) are also generated between 0 and 1 at the beginning of every cycle for each bound kinesin. The cycle of kinesin starts with state [K + MT]. First, if the value of *P*_*D*0_ becomes *r*_*d*0_ before the transition from [K + MT] to [K.ATP + MT]_1_ occurs, then the kinesin unbinds when *P*_*D*0_ is equal to the value of *r*_*d*0_. Otherwise, the chemical state of kinesin changes to [K.ATP + MT]_1_ when P_[*K*+*MT*]_ becomes *r*_*w*_. Then, if the value of *P*_*D*1_ is higher than *r*_*d*1_, then the kinesin unbinds during the diffusion of its free head. Otherwise, the free head moves to the next binding site without unbinding. At this moment, the free head can move to the forward binding site or to the backward binding site. The backward motion of kinesin can be captured by considering the diffusing motion of the kinesin head which is affected by the force acted on the kinesin molecule [25]. In this study, if the probability of backward steps (which is obtained using experimental results [20]) is larger than the random number *r*_*b*_, the kinesin is assumed to move backward. Otherwise, it walks toward the plus end of the MT. When P_[*K*+*MT*]_+*P*_[*K*.*ATP*+*MT*]_2__ becomes *r*_*w*_, the transition from [K.ATP + MT]_2_ to [K.ADP.Pi + MT] occurs. If two or more kinesins are attached on the cargo, the probability distribution of rebinding for every unbound kinesin is also calculated at every time step. First, uniformly distributed random numbers *r*_*a*1_ and *r*_*a*2_ are generated for each unbound kinesin. If the summation of the rebinding probabilities over binding sites (shown in Fig. 5 (g)) are larger than *r*_*a*1_ at a certain time step, the rebinding occurs. Otherwise, the unbound kinesin does not bind to the MT at this time step. If the kinesin is determined to bind at this time step, the rebinding probabilities are normalized with their summation. Then, the normalized rebinding probabilities are cumulated over binding sites. The kinesin binds to the binding site where this cumulative value is larger than *r*_*a*2_.

### Verification of the model

**Fig 8:**
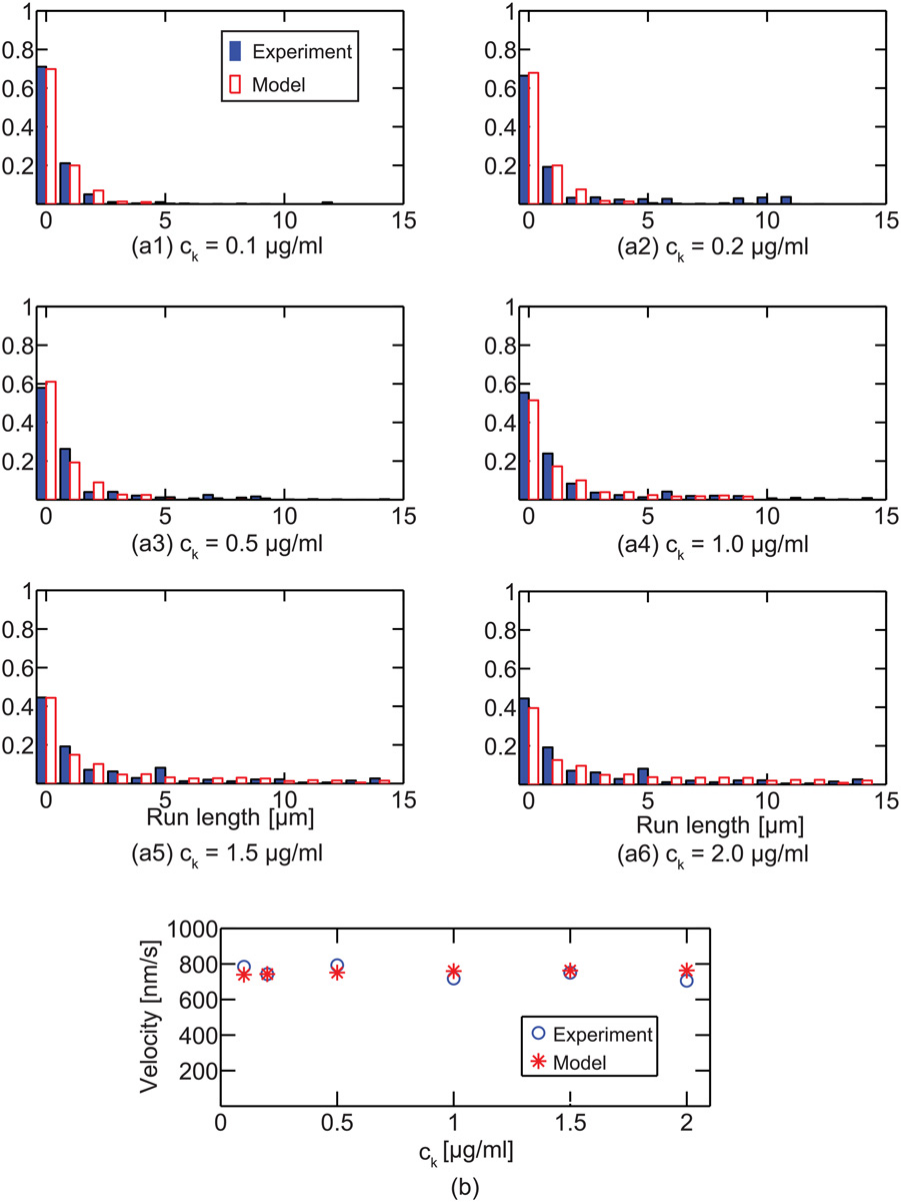
The run length distribution and mean velocity for various concentrations of kinesins are shown. All results are obtained in the absence of loads on the cargo. Whereas the run length increases with *c*_*k*_, the velocity is almost constant for the shown concentrations.

The run length and velocity calculated from the model were compared with the previous experimental data [14]. They mixed beads with kinesins to obtain cargoes coated with kinesins. Different concentrations of kinesins (*c*_*k*_) were used to observe the effects of the number of kinesins attached to single cargoes. Their results are shown in Fig. 8. The results of the model for the used *c*_*k*_ are obtained as follows. The run length distribution and mean velocity of the cargo transported by one to five kinesins were calculated from the model. Then, the weighted averages of these results were calculated by using the probability regarding the number of kinesins (which was proposed in a previous study [14]) for each *c*_*k*_. The run length distribution and mean velocity of the model are similar to the experimental results, as shown in Fig. 8.

## Results

### Unbinding probability

**Fig 9:**
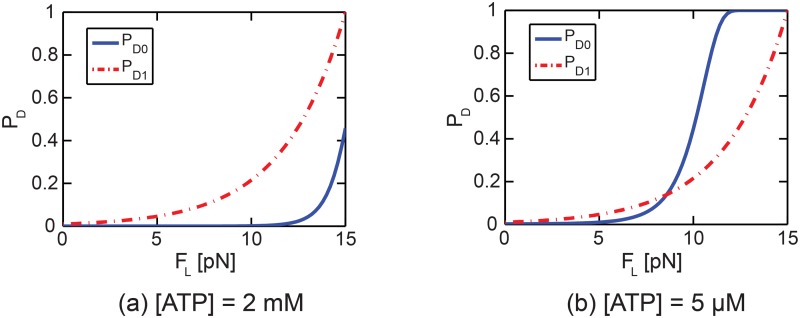
The unbinding probabilities for various resisting loads are presented. The solid line denotes *P*_*D*0_, and the dotted line shows *P*_*D*1_. The unbinding probabilities for [ATP] = 2 mM (a) and 5 *μ*m (b) are shown. Note that *P*_*D*1_ is much higher than *P*_*D*0_ in a wide range of loads for high [ATP].

The unbinding probabilities for the states [K+MT] and [K.ATP + MT]_1_ (i.e., *P*_*D*0_ and *P*_*D*1_) are calculated for various forces applied to single kinesins and for various ATP concentrations, as shown in Fig. 9. The values of *P*_*D*0_ in Fig. 9 are unbinding probabilities when the time *t* in Equation 2 is the average duration of state [K+MT]. When the resisting load is not significant, the duration is very short. Thus, *P*_*D*0_ is close to zero (e.g., for loads *F*_*L*_ between 0 and 5 pN at [ATP] = 5 *μ*m). For large resisting loads, the duration of state [K+MT] is long. Hence, *P*_*D*0_ is large and increases with the load (e.g., for *F*_*L*_ loads between 5 and 12 pN at [ATP] = 5 *μ*m). Also, *P*_*D*0_ exponentially converges to 1 by Equation 2 (e.g., for a load *F*_*L*_ of approximately 12 pN at [ATP] = 5 *μ*m), as shown in Fig. 9 (b).

Note that *P*_*D*1_ is much higher than *P*_*D*0_ for a wide range of forces when the ATP concentration is high. This difference suggests that most kinesin molecules unbind while in the state [K.ATP + MT]_1_ if the ATP concentration is high enough (so that an ATP molecule binds to the kinesin head very fast). This rapid binding of ATP to the kinesin head decreases *P*_*D*0_ significantly. This characterization on the unbinding of kinesins predicts that kinesins mostly unbind when their free heads move to the next binding sites. If large resisting loads act on a kinesin, then the time required to complete one cycle of kinesin is very long. Thus, the interval between steps is also very long for the large loads. Consequently, the time until the unbinding of the kinesin occurs is very long when the load is large. This is a specific kinesin behavior which we refer to as highly loaded behavior (HLB) of kinesins. Because the leading kinesin undergoes the largest resisting force among kinesins, HLB is mostly likely to be observed in the leading kinesin. However, when the resisting load acting on the cargo is very large, then the next or second next leading kinesins also can exhibit HLB together with the first leading kinesin. The effects of HLB on the collective transport are described in the following.

### Highly loaded behavior (HLB) of kinesins for robust intracellular transport: ability to overcome obstacles

**Fig 10:**
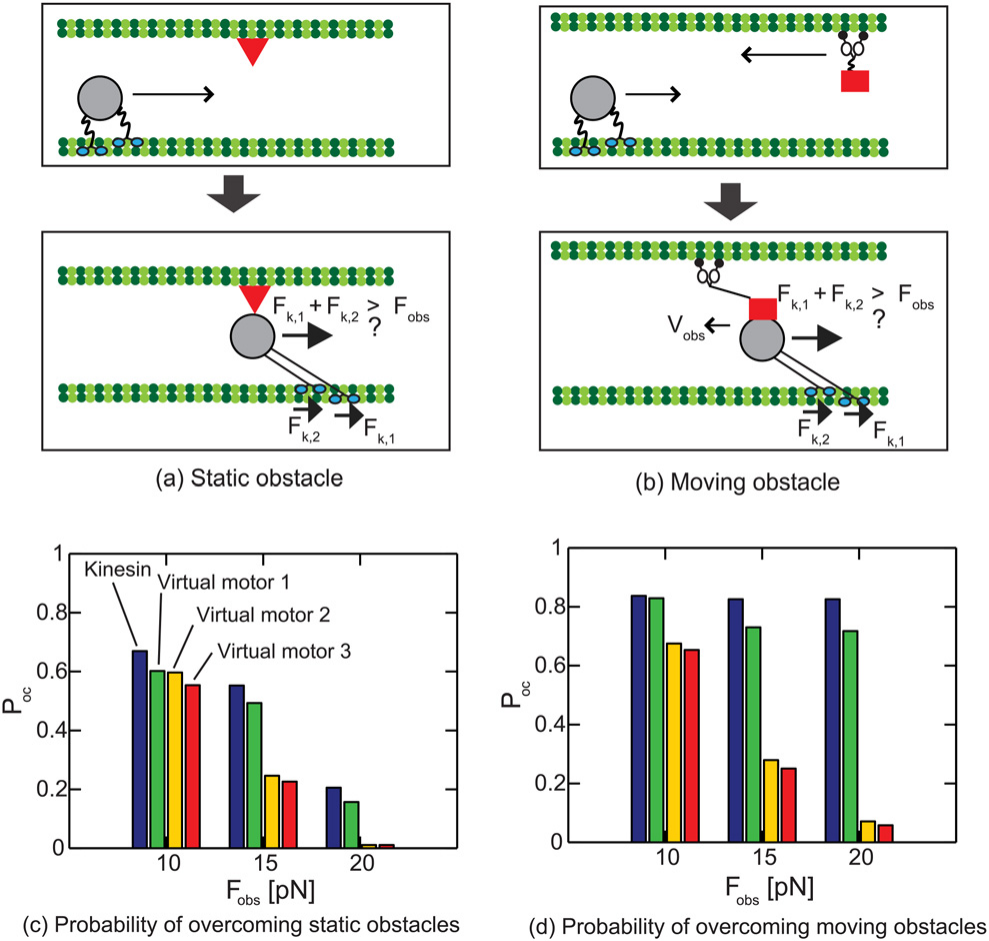
The transport of the cargo in the presence of obstacle is depicted. (a) depicts transport when a static obstacle (shown as a triangle) is on the path of the cargo. (b) shows a moving obstacle (shown as a rectangle) approaching the cargo. The cargo moves backward with velocity of *v*_obs_ due to the moving obstacle. *F*_k,1_ and *F*_k,2_ are forces (acting on the cargo) of two kinesins. The cargo overcomes the obstacle when the sum of those two forces is larger than *F*_obs_. (c) and (d) present the probability *P*_*oc*_ that the cargo overcomes one obstacle without unbinding from the MT. (c) shows the probabilities for static obstacles which require forces of 10, 15, and 20 pN to be overcome it. (d) shows probabilities to overcome moving obstacle when the cargo is retrograded by the obstacle with the velocity of 200 nm/s.

Transport by kinesins can be inhibited by other surrounding particles which act like obstacles. To consider the effect of cellular particles on the motion of the cargo, static and moving obstacles are modeled in this study. First, a static obstacle is located ahead of the cargo, as shown in Fig. 10 (a). When the cargo confronts a static obstacle, the cargo is assumed stuck to the static obstacle until the sum of the forces generated by kinesins exceeds a force *F*_obs_ which is required to overcome the obstacle. Second, the obstacle is assumed to move backward, toward the minus end of the MTs. This motion pushes the cargo backward with a velocity *V*_obs_, as shown in Fig. 10 (b). The retrograde motion due to the moving obstacles also vanishes when the sum of the forces generated by kinesins exceeds a force *F*_obs_.

To check the role of HLB in overcoming obstacles, three virtual motors are created to compare their abilities of overcome obstacles with that of kinesin. The virtual motors are designed by modifying properties of kinesin which are necessary for HLB. First, the virtual motor 1 is modeled so that the unbinding probabilities for three states (i.e., [K+MT], [K.ATP + MT]_1_ and [K.ATP + MT]_2_) are the same. To apply this modification, the unbinding probabilities corresponding to the current force on the kinesin are added together. Then, that summation is divided by three and assigned to the current state so that the motor has equal unbinding probabilities for those three states. Second, the virtual motor 2 has a stepping frequency which is invariant over forces acting on the motor. This modification is accomplished by removing the dependency of the chemical reactions on forces. Third, the virtual motor 3 has both of the changes of virtual motors 1 and 2. Note that the unbinding probability (of a single molecule) per step is the same for all motors, virtual and actual. However, the responses of the teams composed of each type of motors in the presence of obstacle are considerably distinct. The team of two actual kinesins show higher probability *P*_*oc*_ of overcoming one obstacle without dissociating from the MT compared to other teams, as shown in Fig. 10. The difference is remarkable for the team of virtual motors 2. This suggests that the low stepping frequency for high loads is the primary reason for the HLB of kinesin. In addition, if a team of motors confront *n*-obstacles, the overcoming probability decreases to $P_{oc}^{n}$. Then, the cargo transported by virtual motors is not able to reach the final destination, while actual kinesins can resist the interruption of obstacles by using their HLB.

### Run length of transport by several kinesins

**Fig 11:**
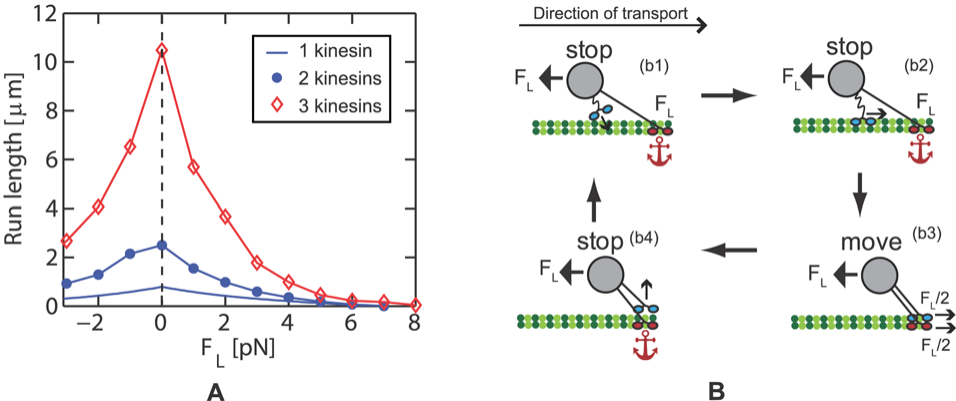
The run length of transport by several kinesins is shown. (a) shows the run length of one, two, and three kinesins for [ATP] = 2 mM. (b) shows an example of HLB when a resisting load of 12 pN acts on the cargo. (b1) The leading kinesin is stationary while waiting for ATP. During this long interval, another kinesin binds to the MT. (b2) It is likely that the distance between the newly bound kinesin and the cargo is less than the length of the cargo linker. Thus, the newly bound kinesin does not have a load. Consequently, the lagging kinesin walks toward the leading one (the anchor) with high velocity. (b3) The two kinesins cooperate to transport their cargo against the large load. (b4) One of the kinesins unbinds and the other kinesin acts as an anchor again.

To obtain run lengths for teams of kinesins, both unbinding and rebinding are considered together with the mechanistic model. At the beginning of the transport, only one kinesin is bound to the MT, and every other kinesin is unbound. Each transport is assumed to be terminated when all kinesins detach from the MT. The run length is defined as the difference in the position of the cargo at the beginning and the position at the termination of the transport. To minimize the error from Monte Carlo simulation, a large number of data is obtained for each load and number of kinesins in the team. The average of the run length over the number of used data converges at about 100 to 150 sets of data. The results presented were obtained using 200 sets to calculate average values. When several kinesins are involved in the transport, the run length of the team increases with the number of kinesins in the team, as shown in Fig. 11 (a). The run length of single kinesins monotonically decreases with the load. However, the run length of a team of kinesins increases with the load when the load is larger than 8 pN.

This interesting feature can be explained by the HLB of kinesins, as shown in Fig. 11 (b). HLB is attributed to properties of the unbinding and stepping frequency. Kinesins mostly unbind when the free heads move to the next binding site. Thus, the unbinding probability for a given time interval increases as the kinesin takes more steps in a given time interval. The stepping frequency of kinesins is decreased by resisting loads. As a consequence, when the high resisting force acts on a kinesin, the kinesin walks slowly and remains bound on the MT for a long time, like an anchor. During this long time, other unbound kinesins attached to the same cargo have time to bind to the MT, as shown in Fig. 11 (b). It is unlikely that large resisting loads, larger than 8 pN, continuously acts on the cargo in cells. However, this HLB can be used in cells to make the transport more robust. Also, we note that the run length will decrease for loads *F*_*L*_ close to *F*_*L*_ = 7 × *n* [pN], where *n* is the number of kinesins attached to the cargo. For those large loads, the cargo will move backward because the kinesins walk backward with high probability when a resisting load close to 7 pN is applied to a single kinesin.

### Velocity of transport by several kinesins

**Fig 12:**
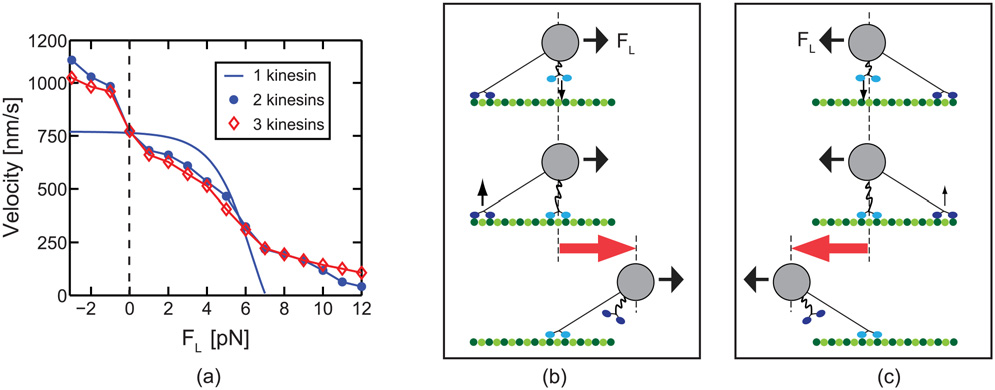
The transport velocity by teams of kinesins is depicted. (a) is the transport velocity by one, two, and three kinesins for [ATP] = 2 mM. (b) and (c) show the effect of the binding and unbinding on the motion of the cargo when the assisting or resisting load is acted on the cargo.

In the absence of load, a single kinesin moves with a velocity of about 800 nm/s [21, 26–28]. The velocity of the transport performed by several kinesins was predicted in this study. For moderate resisting loads, the transport is realized with slower velocity compared to the motion of single kinesins. While the velocity of single kinesins reach a maximum around 800 nm/s at small loads, the motion of a team of kinesins is accelerated by assisting loads, as shown in Fig. 12 (a).

The motion of collective transport can be decelerated or accelerated by binding and unbinding of kinesins. When an assisting load acts on the cargo, the unbound kinesin is likely to bind to a binding site located in front of the other, already bound kinesin as shown in Fig. 12 (b). Consequently, the cargo moves forward a distance longer than just 8 nm (one kinesin step). Hence, the velocity of the cargo increases beyond that of a single kinesin, as shown in Fig. 12 (a). This behavior contrasts single molecule experiments at high ATP concentrations where the velocity is not increased by assisting loads. Also, resisting loads (less than 6 pN) decrease the transport velocity. That is because, for resisting loads, the unbound kinesin rebinds with high probability behind the other, already bound kinesin as shown in Fig. 12 (c). Thus, the transport is decelerated by resisting loads less than 6 pN, as shown in Fig. 12 (a). The velocities for resisting loads higher than 7 pN are larger than the single kinesin velocity. This difference is due to the degree of cooperative work of kinesins for various loads [16]. Due to the slack behavior of kinesins, some bound kineins in a team do not generate forces for transport if the load is small. However, a team is predicted to work in a cooperative fashion for high resisting loads.

## Discussion

When several kinesins are involved in a transport, the characteristics of the transport are significantly affected by the unbinding and rebinding of the kinesins. Novel methods to predict the probability to rebind were presented to reduce the computational effort dramatically. Particularly, the conversion from time average to spatial integration is the key advantage. Note that this approach can be used for other systems which have properties fluctuating with high frequencies. This method revealed that the instant of rebinding depends very weakly on the load. The changes of the run length and of velocity over the load are different for a single kinesin and for a team of kinesins.

The possibility of transport against resisting loads larger than 7 pN per motor implies that the capabilities of a single kinesin can be enhanced by teams of kinesins. To transport a cargo in cells, the cargo needs to navigate in highly viscoelastic cytoplasm which is filled with several particles [29–31]. A single kinesin is not reliable to perform that task for at least two reasons. First, when the resisting load is higher than 7 pN, processivity of single kinesins is not guaranteed because its backward motion occurs frequently. Second, the observed distribution of the run length [10, 32–35] indicates that most of single kinesins detach from the MT before they reach 1 *μ*m. However, a group of kinesins in this study shows comparable velocity toward the plus end of the MT for a broad range of loads.

The velocity of collective transport has three noticeable characteristics compared to the velocity of single kinesins. First, the cargo of several kinesins moves faster than the cargo transported by a single kinesin for assisting loads and slower for resisting loads less than 6 pN. Second, the increase in transport velocity due to assisting loads is larger than the decrease due to resisting loads. Third, the magnitude of the changes in velocity is similar for two and three kinesins. Although the cargo of three kinesins experiences more frequent binding and unbinding events, the distances of the anterograde and retrograde motion of the cargo indicated by red arrows in Figs. 12 (b) and 12 (c) are shorter for three kinesins. These characteristics are also observed in previous experiments. Dujovne et al. [36] applied external forces on MTs in their inverted gliding assay by using an electric field. In their experiments, the velocity increases or decreases due to assisting or resisting loads. Also, the change in the velocity is larger for assisting loads, like our results. The low and medium density of kinesin shows a similar velocity change over the electric field. This is also consistent with the velocity predictions in this study.

The team operation of kinesins also prevents the early termination of the transport by obstacles in cells. The interference between the cargo and the obstacles can result in significant loads on the cargo. Especially in axons where several MTs are aligned, the motion of the cargo can be affected by static obstacles such as MT associated proteins, or tangles of the proteins [37]. Also, the cargo could encounter other cargoes transported by different motor proteins such as dyneins which move reverse to the walking direction of kinesin. The cargoes transported toward the minus end of MTs act as moving obstacles to the anterograde transport of kinesins. The huge load resulting from the static and moving obstacles is fatal to the transport if the cargo is transported by a single kinesin. However, if several kinesins are attached to the cargo, one or more kinesins function as temporary anchors until the transient load vanishes. Thus, kinesins are able to continue and complete their task as a team. The comparison of transport by actual kinesins and virtual motors suggests that the reduction in velocity of kinesins for high resisting loads is necessary to maintain cellular transport in the presence of obstacles. When the motion of the cargo is blocked by obstacles, the leading kinesin acts like an anchor as its velocity decreases. Thus, time is available for the other kinesins in the team to cooperate and overcome the interruption due to the obstacle. Together with the velocity of the transport by several kinesins, the HLB suggests that cells utilize teams of kinesins for the (harsh) cellular transport.

## Supporting Information

S1 Text Supplementary information describes details on the mechanistic model. (PDF)

S1 Fig Probability of chemical states is depicted. (a) shows the changes of the states of kinesin over time in the absence of a load. The thin solid line is the probability of the state [K+MT], and the dotted line and the thick line denote the probability of the states [K.ATP + MT]_2_ and [K.ADP.Pi + MT]. (b) shows the cumulative probabilities. They are used to determine the instant of the transition between states.
(EPS)

S2 Fig Backward step cycle is depicted. ATP binds to the leading head (a); next, the trailing head moves forward due to the conformational change (b); due to the large resisting load acting on the cargo linker, the free head diffuses back to its original site; then, the head attaches to the MT by releasing ADP (c), and ATP is hydrolyzed in the other head (d).
(EPS)

S3 Fig Velocities of single kinesins for high and low ATP concentrations are shown. (EPS)
