# Supplementary material for: Highly Loaded Behavior of Kinesins Increases the Robustness of Transport Under High Resisting Loads
Source: PLoS Comput Biol. 2015 Mar 3;11(3):e1003981. doi: 10.1371/journal.pcbi.1003981 (PMC4347983; doi:10.1371/journal.pcbi.1003981)
Supplement: S1 Text — (PDF) [file pcbi.1003981.s001.pdf]

Supporting information: Highly loaded behavior of kinesins increases the robustness of transport under high resisting loads

Woochul Nam, Bogdan I. Epureanu*,
Department of Mechanical Engineering, University of Michigan, Ann Arbor, Michigan 48109-2125, USA
∗ E-mail: epureanu@umich.edu

Mechanistic model of kinesin

The mechanistic model considers the forces acting in every domain of a kinesin protein, and the effects of forces (stresses) on the chemical reaction between the kinesin heads and ATP molecules.

Forces in kinesin and cargo

The force F_c acting on the cargo is obtained by adding every force $F_{\mathrm{k},i}$ generated by each kinesin i and the external load F_L as

$$F_c = -F_L + \sum_{i=1}^{N} F_{\mathrm{k},i}. \tag{S1}$$

The external load is expressed with minus because the positive direction is considered toward the minus end of MT. Since the Reynolds number regarding the motion of the cargo is very small, the position of the cargo can be determined using Stokes' law as

$$x_c(t) = x_c(0) + \frac{1}{6\pi r_c \eta} \int_0^t F_c(\tau) d\tau. \tag{S2}$$

where r_c is the radius of the cargo and η denotes the viscosity of the fluid. The viscosity of water is used in this study.

The cargo linker is composed of two coils which are folded onto each other. The coil contains about 700 amino acids [1]. The distance between adjacent amino acids in the coil along its axis is approximately 0.15 nm [2]. Also, the pitch angle of the coiled coil is about 20° [3]. Thus, the length of the cargo linker can be estimated to be approximately 100 nm (because $0.15 \times 700 \times cos(20°) = 98.7$). Thus, the ability of the cargo linker to support forces is assumed to be negligible when the distance between the cargo and the neck is smaller than 100 nm. Note that this is the distance between the ends of the cargo linker. The same distance, measured along the MT is smaller due to geometric effects (2 or 3D). The value of the distance between the end points along the MT where loads start to be transferred is smaller than 100 nm and is denoted by L_c. When the radius of the cargo is 50 nm, the value of L_c can be calculated as 94 nm using the geometric relation suggested by Fehr et al. [4]. Thus, the force transferred to the neck of the ith kinesin via its cargo linker is obtained as

$$F_{\mathrm{k},i} = \begin{cases} K_c(x_{n,i} - x_c - L_c) & \text{if } x_{n,i} \geq x_c + L_c, \\ K_c(x_{n,i} - x_c + L_c) & \text{if } x_{n,i} \leq x_c - L_c, \\ 0 & \text{otherwise,} \end{cases} \tag{S3}$$

While a kinesin head waits for ATP to bind, it is assumed that the whole force on the neck is transferred to the forward head and the other head does not have any load. Experiments support this assumption [5, 6]. After the diffusion of the free head to the next binding site, both heads are strongly bound to the MT. Thus, the force on the neck is transferred through both neck linkers. The force transferred by the neck linkers to the neck is calculated as

$$F_{n,i} = \begin{cases} K_n(x_{fh,i} - x_{n,i}) & \text{if the kinesin is in state [K+MT],} \\ 2K_n\left(\frac{x_{fh,i}+x_{bh,i}}{2} - x_{n,i}\right) & \text{if the kinesin is in state } [\text{K.ATP} + \text{MT}]_2, \end{cases} \tag{S4}$$

Both $F_{\mathrm{k},i}$ and $F_{n,i}$ are applied at the neck. The size of the neck is assumed negligible. Thus, these two forces have to balance. The positions of the cargo and the necks of all kinesins are obtained at every time step as the values which satisfy this force equilibrium, Equations S1 and S2.

Chemical reaction

The chemical reaction is described using the transition between the chemical states (i.e., [K+MT], $[\mathrm{K.ATP}+\mathrm{MT}]_1$, $[\mathrm{K.ATP}+\mathrm{MT}]_2$, and $[\mathrm{K.ADP.Pi}+\mathrm{MT}]$) of a kinesin molecule. The probabilities of states are determined by the transition rates. Probabilities satisfy

$$\begin{aligned}
&\frac{d}{dt}P_{[\mathrm{K+MT}]} = -k_{1f}[\mathrm{ATP}]P_{[\mathrm{K+MT}]} + k_{1b}P_{[\mathrm{K.ATP+MT}]_1}\\
&\frac{d}{dt}P_{[\mathrm{K.ATP+MT}]_1} = k_{1f}[\mathrm{ATP}]P_{[\mathrm{K+MT}]} - k_{1b}P_{[\mathrm{K.ATP+MT}]_1} - k_{2f}P_{[\mathrm{K.ATP+MT}]_2},\\
&\frac{d}{dt}P_{[\mathrm{K.ADP.Pi+MT}]} = k_{2f}P_{[\mathrm{K.ATP+MT}]_2},\\
&P_{[\mathrm{K.ATP+MT}]_1} = P_{[\mathrm{K.ATP+MT}]_2}
\end{aligned} \tag{S5}$$

where $P_{[\mathrm{K+MT}]}$, $P_{[\mathrm{K.ATP+MT}]_1}$, $P_{[\mathrm{K.ATP+MT}]_2}$ and $P_{[\mathrm{K.ADP.Pi+MT}]}$ denote the probabilities of chemical states of kinesin shown in Figure 2. Parameters k_{1f}, k_{1b}, and k_{2f} are the transition rates between the states. If the ith cycle starts at t_i, the probability of the current state during the cycle is obtained by solving Equation S5 with the initial conditions $P_{[\mathrm{K+MT}]}(t_i) = 1$, and $P_{[\mathrm{K.ATP+MT}]_1}(t_i) = P_{[\mathrm{K.ATP+MT}]_2}(t_i) = P_{[\mathrm{K.ADP.Pi+MT}]}(t_i) = 0$. Figure S1 shows the probabilities of the bound states over time. The two curves in Figure S1 (b) show the two probabilities, $P_{[\mathrm{K+MT}]}(t)$ and $P_{[\mathrm{K+MT}]}(t) + P_{[\mathrm{K.ATP+MT}]_2}(t)$.

To determine the instants of transitions, a uniformly distributed random number (r_w) between 0 and 1 is generated. When $P_{[K+MT]}$ becomes r_w, the transition from [K+MT] to $[\mathrm{K.ATP}+\mathrm{MT}]_1$ occurs. Then, the next transition from $[\mathrm{K.ATP}+\mathrm{MT}]_2$ to $[\mathrm{K.ADP.Pi}+\mathrm{MT}]$ occurs when $\mathrm{P}_{[\mathrm{K+MT}]} + \mathrm{P}_{[\mathrm{K.ATP+MT}]_2}$ reaches r_w.

The transition rate regarding the dissociation of an ATP molecule from the forward head is assumed to depend on the stresses on the kinesin head as

$$k_{1b} = k_{1b,0}\,\exp\frac{\frac{1}{2}\kappa(\frac{F_{\mathrm{k}}}{\kappa} - \Phi_c)^2}{k_B T}, \tag{S6}$$

where Φ_c and κ are parameters of the model [7].

Backward step

If a large load acts on the cargo, kinesins walk toward the minus end of the MT as well as toward the plus end [8, 9]. Because the run length and velocity of the cargo depend on the backward motion of kinesins, the backward motion is also considered in this study. In the model we adopted [10], the kinesin walks backward when a large load acts on the kinesin molecule because the free head diffuses back to its original site, as shown in Figure S2. The model parameters are obtained using experimental results obtained by Carter et al. [8]. They observed that the probability of backward steps increases exponentially with the external load.

To model this process we use the following algorithm. When the ATP is bound to the head of a kinesin, the probability of backward steps corresponding to the force on the kinesin is calculated, and a uniformly distributed random number between 0 and 1 is generated. If the random number is larger than the probability of a backward step, then the free head of the kinesin diffuses to the forward binding site. Otherwise, the free head moves to the binding site behind the fixed head.

Parameters

Table S1 shows the values of the parameters of the mechanistic model. Note that the rate constants of the model in the absence of external loads are similar to the values obtained in the experiment of ATPase [11]. K_c is defined as the experimentally obtained value [12]. In this study, the neck linker is assumed to behave like a linear spring. Thus, the value of K_n is determined so that the linear spring has the same strain energy with the energy of the worm like chain model in [13]. The parameters regarding chemical rate constants (i.e., k_{1f}, k_{1b}, and k_{2f}) of set A are experimentally measured values in the absence of load [11, 14]. The other values (i.e., Φ_c and κ) are obtained by fitting to the measured velocity from the single molecule experiment [15]. When ATP is high, these values predict the velocity similar to experiments, as shown in Figure S3 (a) below. However, the velocity of the model does not match to the experimental data when ATP is low, as shown in Figure S3 (b) below. Thus, parameters of set B are obtained by varying the chemical reaction constants when fitting the model to the experimental force velocity curve. Note that the value of k_{1b} in the absence of load (i.e., $k_{1b,0}$) is 70 s^{-1} in set A, and 59.1 s^{-1} in set B. The difference comes from the dependence on load which is modeled with Equation S6. The chemical rate constants in the absence of loads of set B are similar to the experimentally measured values by Gilbert et al. [11, 14]. Also, velocities predicted from set B are similar to the experimentally measured velocity for both high and low ATPs, as shown in Figure S3 (a) and (b). Hence, the parameters in set B were used.

Figures Legends

Figure S1. Probability of chemical states is depicted. (a) shows the changes of the states of kinesin over time in the absence of a load. The thin solid line is the probability of the state [K+MT], and the dotted line and the thick line denote the probability of the states $[\mathrm{K.ATP} + \mathrm{MT}]_2$ and $[\mathrm{K.ADP.Pi} + \mathrm{MT}]$. (b) shows the cumulative probabilities. They are used to determine the instant of the transition between states.

Figure S2. Backward step cycle is depicted. ATP binds to the leading head (a); next, the trailing head moves forward due to the conformational change (b); due to the large resisting load acting on the cargo linker, the free head diffuses back to its original site; then, the head attaches to the MT by releasing ADP (c), and ATP is hydrolyzed in the other head (d).

Figure S3. Velocities of single kinesins for high and low ATP concentrations are shown.

Tables

Table S1. The values of parameters of the mechanistic model.

Parameter	Set A	Set B
K_c [pN/nm]	0.3	0.3
K_n [pN/nm]	3.8	3.8
k_{1f} [μM^{-1}s^{-1}]	2	2.385
$k_{1b,0}$ [s^{-1}]	43.0	32.921
k_{2f} [s^{-1}]	100	98.875
Φ_c [nm]	1.009	1.2114
κ [pN/nm]	3.964	3.302

References

1. Hirokawa N, Noda Y (2008) Intracellular transport and kinesin superfamily proteins, kifs: structure, function, and dynamics. Physiological Reviews 88: 1089–1118.

2. Lupas AN, Gruber M (2005) The structure of α-helical coiled coils. Advances in Protein Chemistry 70: 37–38.

3. Lupas A (1996) Coiled coils: new structures and new functions. Trends in Biochemical Sciences 21: 375–382.

4. Fehr A, Gutiérrez-Medina B, Asbury C, Block S (2009) On the origin of kinesin limping. Biophys Journal 97: 1663–1670.

5. Asenjo AB, Sosa H (2009) A mobile kinesin-head intermediate during the atp-waiting state. Proceedings of the National Academy of Sciences, USA 106: 5657–5662.

6. Guydosh NR, Block SM (2009) Direct observation of the binding state of the kinesin head to the microtubule. Nature 461: 125–128.

7. Hendricks A, Epureanu B, Meyhöfer E (2008) Mechanistic mathematical model of kinesin under time and space fluctuating loads. Nonlinear Dynamics 53: 303–320.

8. Carter N, Cross R (2005) Mechanics of the kinesin step. Nature 435: 308-312.

9. Nishiyama M, Higuchi H, Yanagida T (2002) Chemomechanical coupling of the forward and backward steps of single kinesin molecules. Nature Cell Biology 4: 790–797.

10. Hyeon C, Klumpp S, Onuchic JN (2009) Kinesins backsteps under mechanical load. Physical Chemistry Chemical Physics 11: 4899–4910.

11. Gilbert SP, Webb MR, Brune M, Johnson KA (1995) Pathway of processive atp hydrolysis by kinesin. Nature 373: 671.

12. Coppin C, Finer J, Spudich J, Vale R (1996) Detection of sub-8-nm movements of kinesin by high-resolution optical-trap microscopy. Proceedings of the National Academy of Sciences, USA 93: 1913.

13. Hariharan V, Hancock WO (2009) Insights into the mechanical properties of the kinesin neck linker domain from sequence analysis and molecular dynamics simulations. Cellular and Molecular Bioengeering 2: 177–189.

14. Gilbert SP, Moyer ML, Johnson KA (1998) Alternating site mechanism of the kinesin atpase. Biochemistry 37: 792–799.

15. Visscher K, Schnitzer M, Block S (1999) Single kinesin molecules studied with a molecular force clamp. Nature 400: 184-189.
